# Serum lysophosphatidylcholines to phosphatidylcholines ratio is associated with symptomatic responders to symptomatic drugs in knee osteoarthritis patients

**DOI:** 10.1186/s13075-019-2006-8

**Published:** 2019-11-06

**Authors:** Guangju Zhai, Jean-Pierre Pelletier, Ming Liu, Edward W. Randell, Proton Rahman, Johanne Martel-Pelletier

**Affiliations:** 1 Discipline of Genetics, Faculty of Medicine, Craig L Dobbin Genetics Research Centre, Memorial University of Newfoundland, St. John’s, NL A1B 3V6 Canada; 2 Osteoarthritis Research Unit, University of Montreal Hospital Research Centre (CRCHUM), Montreal, QC Canada; 3 Department of Laboratory Medicine, Faculty of Medicine, Memorial University of Newfoundland, St. John’s, NL Canada; 4 Discipline of Medicine, Faculty of Medicine, Memorial University of Newfoundland, St. John’s, NL Canada

**Keywords:** Knee osteoarthritis, Serum biomarkers, Patient prioritization, NSAIDs, Licofelone

## Abstract

**Background:**

Identification of the optimal treatment for a given patient is of paramount importance. This is of particular relevance in osteoarthritis (OA) because of the high prevalence of the disease, extensive heterogeneity of the disease, and need for long-term treatment. The aim of the study was to examine whether serum lysophosphatidylcholines (lysoPCs) to phosphatidylcholines (PCs) ratio can predict clinical response to licofelone and naproxen treatments in symptomatic knee OA patients.

**Methods:**

One hundred fifty-eight OA patients who completed the study according to protocol (ATP) of a previous 24-month clinical trial cohort comparing the effect of licofelone vs. naproxen in symptomatic knee OA patients were included. Symptomatic responses to either treatments were classified according to the OARSI-OMERACT criteria based on the WOMAC scores at 24 months. Total concentrations of PCs and lysoPCs were measured in the serum samples collected before the initiation of the treatments, and the lysoPCs to PCs ratio was calculated. Student’s *t* test was utilized to compare the difference in the ratio of lysoPCs to PCs between the symptomatic responders and non-responders. Logistic regression was utilized to adjust for the potential confounders. Receiver operating characteristic (ROC) analysis was performed to identify the optimal cutoff of the ratio for prediction.

**Results:**

Data showed that 61.4% of the patients symptomatically responded to licofelone and naproxen and 38.6% were deemed as therapeutic failures (non-responders). There was no difference in responders between licofelone and naproxen (*p* = 0.87). Responders had a significantly higher lysoPCs to PCs ratio than non-responders (0.097 ± 0.003 vs. 0.085 ± 0.003; *p* = 0.006). Patients with a ratio greater than the optimal cutoff of 0.088 had 2.93 times more likely to respond to licofelone and naproxen (*p* = 0.002).

**Conclusions:**

Serum lysoPCs to PCs ratio is a marker for response to licofelone and naproxen and may aid in the personalized treatment to knee OA.

## Background

Osteoarthritis (OA) is the most common form of arthritis, affecting about 10% of the world’s population and about half of the population aged 60 years or older [1]. This disease demonstrates a major source of joint pain and disability [2] and imposes a substantial socioeconomic burden on society with a cost estimate of between 1 and 2.5% of gross domestic product [3]. However, there is no cure for it yet. At present, the primary goal of treatment is symptom management.

Non-steroidal anti-inflammatory drugs (NSAIDs) are widely used effective agents for symptomatic management for OA patients. NSAIDs exert their effects by inhibiting biosynthesis of prostaglandins (PGs) by acting on the cyclooxygenase enzymes (COX). However, NSAIDs differ widely in their chemical composition and class, and the response to them varies among patients. For example, while a clinical trial demonstrated a significant mean difference in pain relief between etoricoxib and placebo in knee OA patients, only 50% of patients achieved at least 50% pain relief for 60 mg etoricoxib [4]. The study showed that at least three to four patients were needed to treat with etoricoxib in order for one patient to achieve at least 50% pain relief [4]. Licofelone is an anti-inflammatory and dual inhibitor of COX (both COX-1 and COX-2) and 5-lipoxygenase (5-LOX) having both analgesic and anti-inflammatory properties [5] and causing little or no damage to the gastric mucosa than NSAIDs [6]. Similar to NSAIDs, response to it also varies among patients. This is not surprising given that OA is known as a heterogeneous disease, and the pain related to this disease can be classified into many different types of pain such as inflammatory, weight-bearing, multisite, and central. It is, therefore, important to develop tools that can identify patients who will respond to a symptomatic treatment. Biomarkers could have the potential for this purpose.

Recent application of metabolomics in OA research has identified several metabolic markers for OA, and lysophosphatidylcholines (lysoPCs) to phosphatidylcholines (PCs) ratio is a very promising one [7, 8]. Given that the conversion of PCs to lysoPCs releases polyunsaturated fatty acids including arachidonic acid, linoleic acid, and linolenic acid, which are precursors of bioactive molecules including eicosanoids, prostanoids, and endocannabinoids involving a variety of physiological processes such as inflammatory processes [9], we hypothesized that the lysoPCs to PCs ratio can predict the response to a symptomatic drug including an NSAID. To test this hypothesis, we used data from a phase III clinical trial of patients with symptomatic knee OA comparing the effect on cartilage volume loss of treatment with licofelone vs. the NSAID naproxen [10].

## Patients and methods

### OA patients

This post hoc study used a cohort from a previously published phase III clinical trial of patients with symptomatic knee OA comparing the effect on cartilage volume loss of oral treatment with licofelone (200 mg twice a day) vs. naproxen (500 mg twice a day) [10]. The patients (*n* = 158) selected were those who completed the study according to protocol (ATP) [10–12], had serum collected at baseline, and WOMAC questionnaire data at baseline and 24 months. Briefly, patients with primary symptomatic knee OA of the medial tibiofemoral compartment, diagnosed according to the American College of Rheumatology (ACR) criteria [13], were recruited from outpatient rheumatology clinics. Patients had a pain level of no less than 40 mm on the visual analog scale, radiographic OA grade 2–3 on the Kellgren-Lawrence (KL) scale, and at least one of the following three risk factors for increased risk of progression: body mass index (BMI) > 30 kg/m^2^, presence of Heberden’s nodes, or female gender. The original study protocol was approved by IRB Institutional Review Board Services, Toronto, ON, Canada, and the institutional review board of the Centre hospitalier de l’Université de Sherbrooke (Sherbrooke, QC, Canada), and all patients gave their oral and written informed consent to participate, including permission for the use of serum to be collected throughout the study for biomarker studies.

### Symptomatic responder assessment

Symptomatic responders were defined according to the OARSI-OMERACT criteria [14]. Patients were classified as responders if they met the following criteria: 50% improvements either in WOMAC pain or function score between baseline and at 24 months and absolute change was ≥ 20 on the 100 scale. For those who did not meet the above criteria, they were classified as responders if they met at least two of three of the following: ≥ 20% pain relief and absolute change ≥ 10; ≥ 20% function improvement and absolute change ≥ 10; and ≥ 20% improvement on global WOMAC score and absolute ≥ 10.

#### Serum metabolic profiling

Metabolic profiling on the serum samples collected at baseline after overnight fasting was performed as previously described [8] using Biocrates *AbsoluteIDQ® p180 kit*. The assay measured 11 different lysoPCs and 73 PCs per sample. The serum concentration data on these lysoPCs and PCs were retrieved from the database, and the total concentration of the lysoPCs and PCs was calculated as the sum of all the different species in each class. The ratio was then derived by total lysoPC concentration divided by total PC concentration and used in the analysis.

### Statistical analysis

The distribution of the lysoPCs to PCs ratio was checked; one subject was identified as an outlier because the ratio was greater than 3 standard deviation (SD) from the mean and removed from the subsequent analysis. Student’s *t* test was utilized to compare the difference in the ratio of lysoPCs to PCs between the symptomatic responders and non-responders. Logistic regression was utilized to examine the potential confounding effects of age, sex, BMI, and treatments. Receiver operating characteristic (ROC) analysis was performed to examine the prediction power of the ratio for symptomatic responders, and the maximizes sensitivity and specificity simultaneously (MaxSpSe) method was used in the OptimalCutpoints R package to identify the optimal cutoff value for the prediction of responders. The analyses were done in STATA/ST 11.2 and R version 3.5.0.

## Results

One hundred fifty-eight symptomatic knee OA patients were included. Based on the OARSI-OMERACT criteria [14], 61.4% were classified as symptomatic responders to licofelone and naproxen and 38.6% were non-responders. Seventy-nine patients were treated with licofelone and 79 with naproxen. There was no difference in the symptomatic responders between licofelone and naproxen treatments (Table 1); thus, further analysis used the entire ATP cohort. The responders were not associated with age, sex, and baseline BMI (Table 1).

**Table 1:** Patient characteristics

Variables	Responders (*n* = 97)	Non-responders (*n* = 61)	*p* value
Age (years)	60.6 ± 0.8	60.9 ± 1.0	0.80
BMI (kg/m^2^)	31.6 ± 0.6	32.2 ± 0.7	0.50
Sex (% female)	68.0	70.5	0.75
Treatments (licofelone and naproxen) (% for licofelone)	49.5	50.8	0.87

Numbers are either mean ± SD or percentage

**Fig. 1:**
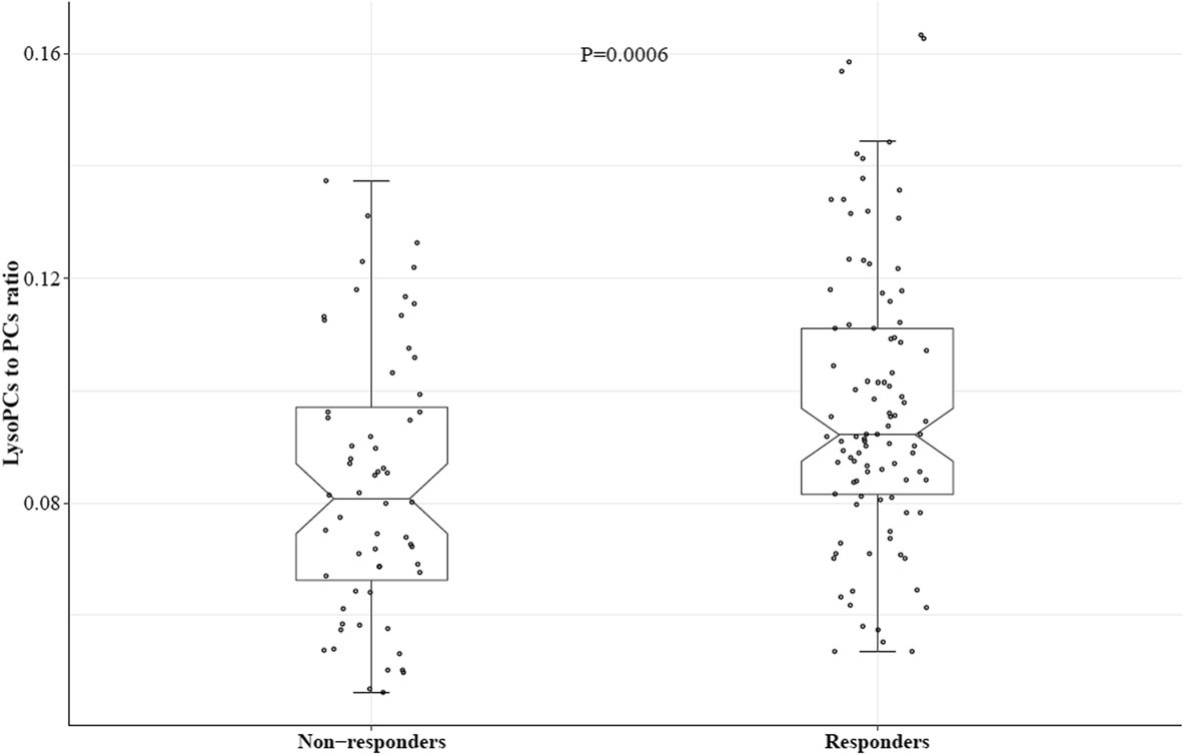
Boxplots for serum lysoPC to PC ratio in symptomatic responders and non-responders to licofelone and naproxen in symptomatic knee OA patients. Note: one outlier was excluded in the analysis, and the total number of patients included was 157. lysoPCs are the total serum concentration of all lysophosphatidylcholines; PCs are the total serum concentration of all phosphatidylcholines

**Fig. 2:**
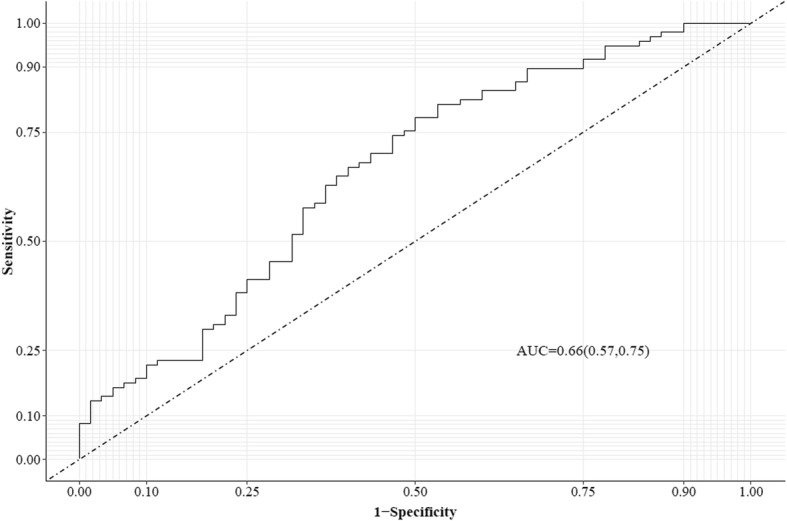
Receiver operating characteristic (ROC) curve for serum lysoPCs to PCs ratio for predicting responders to licofelone and naproxen in symptomatic knee OA patients. lysoPCs are the total serum concentration of all lysophosphatidylcholines; PCs are the total serum concentration of all phosphatidylcholines

One patient as an outlier was excluded in the subsequent analysis. Symptomatic responders had a significantly higher serum lysoPCs to PCs ratio than non-responders (0.097 ± 0.003 vs. 0.083 ± 0.003; *p* = 0.0006) (Fig. 1). ROC curve analysis showed that the ratio had an AUC of 0.66 (95% CI 0.57, 0.75) to distinguish symptomatic responders from non-responders (Fig. 2). The optimal cutoff was determined as 0.088 by the OptimalCutpoints R package with the MaxSpSe method. Using the optimal cutoff, patients with a ratio of ≥ 0.088 had 2.93 times (95% CI 1.50, 5.70; *p* = 0.002) more likely to respond to licofelone and naproxen than those with a ratio of < 0.088. The association between the lysoPCs to PCs ratio and symptomatic responders virtually remained the same after adjustment for age, sex, BMI, and treatments.

## Discussion

NSAIDs are the first choice for OA patients to manage their joint pain because of their robust efficacy and long history of clinical use. However, NSAIDs are a chemically heterogeneous group of compounds, and the response to them varies among patients, whereas licofelone, a dual inhibitor of both COX and 5-LOX, was also shown to have symptomatic benefit in OA patients [5].

Our data demonstrated, for the first time, that the metabolomic product serum lysoPCs to PCs ratio is associated with responders of OA patients to the symptomatic treatments licofelone and naproxen. Such data provides a personalized medicine tool for clinicians when treating joint pain for OA patients.

Previously, we reported that the lysoPCs to PCs ratio in the plasma was associated with knee OA risk [7]. In which the ratio of 0.09 was the optimal cutoff to distinguish advanced knee OA patients from healthy individuals [7]. Moreover, we found that people with a ratio greater than or equal to this optimal cutoff were 2.3 times more likely to undergo total joint replacement surgery due to primary OA over 10 years [7]. Interestingly, the same cutoff number was found in the current study as the optimal one to distinguish knee OA patients who symptomatically responded to the treatment with licofelone and naproxen from those who did not, suggesting the optimal cutoff can be generalized to other population.

Increased lysoPCs to PCs ratio indicates a higher activity of the conversion of PCs to lysoPCs. In turn, such activity leads to the release of long-chain polyunsaturated fatty acids, like arachidonate, then to the activation of eicosanoid pathways and production of bioactive compounds such as PGs leading to OA joint symptoms. Thus, an increased lysoPCs to PCs ratio points to an elevated inflammatory process within the body. Given that both products, licofelone and naproxen, target the PGs inhibition, it is not surprising that a higher ratio can predict symptomatic responders. This could imply that responders had a higher level or more predominant level of the inflammatory process than the non-responders, thus more likely to benefit from the treatments that target on inflammation process. In this line of thought, it has also been reported that this ratio is an indicator of the activity of another arthritis disease, rheumatoid arthritis (RA), and can monitor the anti-inflammatory effects of TNF-⍺ in those patients [15]. This latter study also demonstrated similar results with either serum or synovial fluid. These data with the findings in the current study suggest that the lysoPCs to PCs ratio might not be disease-specific but rather inflammation-specific. However, further studies are needed to confirm this assumption.

The limitation of the study was that all the study participants were obese. While the observed association virtually remained the same after adjustment for BMI, further studies are needed to examine whether the current findings can apply to non-obese patients.

In conclusion, our data suggest that serum lysoPCs to PCs ratio could be considered as a personalized medicine tool for prioritizing OA patients receptive to respond to a symptomatic drug.

## Data Availability

The datasets used and/or analyzed during the current study are available from the corresponding author on reasonable request.

## Abbreviations

5-LOX: 5-Lipoxygenase
ACR: American College of Rheumatology
ATP: According to protocol
BMI: Body mass index
COX: Cyclooxygenase enzymes
KL: Kellgren-Lawrence
lysoPCs: Lysophosphatidylcholines
MaxSpSe: Maximizes sensitivity and specificity simultaneously
NSAIDs: Non-steroidal anti-inflammatory drugs
OA: Osteoarthritis
OARSI: Osteoarthritis Research Society International
OMERACT: Outcome Measures in Rheumatology
ON: Ontario
PCs: Phosphatidylcholines
PG: Prostaglandins
RA: Rheumatoid arthritis
ROC: Receiver operating characteristic
SD: Standard deviation
TNF-⍺: Tumor necrosis factor alpha
WOMAC: Western Ontario and McMaster Universities Osteoarthritis Index
